# HIV-1 Nef: at the crossroads

**DOI:** 10.1186/1742-4690-5-84

**Published:** 2008-09-22

**Authors:** John L Foster, J Victor Garcia

**Affiliations:** 1 Department of Internal Medicine, Division of Infectious Diseases, University of Texas Southwestern Medical Center, Dallas, TX 75390

## Abstract

The development of anti-virals has blunted the AIDS epidemic in the Western world but globally the epidemic has not been curtailed. Standard vaccines have not worked, and attenuated vaccines are not being developed because of safety concerns. Interest in attenuated vaccines has centered on isolated cases of patients infected with HIV-1 containing a deleted *nef *gene. Nef is a multifunctional accessory protein that is necessary for full HIV-1 virulence. Unfortunately, some patients infected with the *nef*-deleted virus eventually lose their CD4^+ ^T cells to levels indicating progression to AIDS.

This renders the possibility of an attenuated HIV-1 based solely on a deleted *nef *remote. In this review we discuss the knowledge gained both from the study of these patients and from in vitro investigations of Nef function to assess the possibility of developing new anti-HIV-1 drugs based on Nef. Specifically, we consider CD4 downregulation, major histocompatibility complex I downregulation, Pak2 activation, and enhancement of virion infectivity. We also consider the recent proposal that simian immunodeficiency viruses are non-pathogenic in their hosts because they have Nefs that downregulate CD3, but HIV-1 is pathogenic because its Nef fails to downregulate CD3. The possibility of incorporating the CD3 downregulation function into HIV-1 Nef as a therapeutic option is also considered. Finally, we conclude that inhibiting the CD4 downregulation function is the most promising Nef-targeted approach for developing a new anti-viral as a contribution to combating AIDS.

## Introduction

The brutal attack on humanity by HIV-1 has proven to be distressingly difficult to counter. The best results at blunting the epidemic have been the development of anti-retrovirals (ARVs) that inhibit crucial HIV-1 functions. Unfortunately, the unique ability of HIV-1 to mutate and adapt [1,2] requires multiple drug treatments that are limited in their application by their side effects and their expense. Topically applied microbicides offer the possibility of prevention, but similar problems of toxicity, expense, and effective application apply here as well as with ARVs [3,4]. Vaccines have been a total failure and future prospects are dim [5-8].

Well into the third decade of HIV-1 research the likelihood of finding an Achilles' heel for HIV-1 is remote. The virus is too highly adapted from its successful 70 year contest with the human immune system [9,10]. Accumulating small victories are the probable long term course for significantly curtailing the epidemic. Effective microbicides are desperately needed for vaginal pre-exposure prophylaxis and post-exposure prophylaxis. New ARVs that inhibit an increasing number of viral processes are critical for treating already infected individuals. ARVs are potentially useful in prophylaxis as well. In this case topically applied drugs would ideally be different from drugs used for treating HIV-1 since topical application could lead to resistant strains of HIV-1 [3,4]. Therefore, all possible targets for countering HIV-1 need to be considered. Given its central role in HIV pathogenesis, in this article we consider Nef as a potential anti-viral target for preventing or at least delaying pathogenesis.

Ironically, the overwhelming focus for a Nef-based therapeutic intervention has been the investigation of a *nef*-deleted attenuated virus vaccine. This interest resulted from a small number of cases of long term non-progressors (LTNP) whose viruses have irretrievable deletions in the *nef *gene [11-14]. Unfortunately, some individuals infected with the *nef*-deleted virus are slow progressors (SP) rendering a *nef*-deleted attenuated vaccine too dangerous. We will not review this aspect of the Nef field in detail since an excellent review has been recently published on the most important of these cases- the Sydney Blood Bank Cohort (SBBC) [15]. We will discuss several aspects of SBBC and other cases that shed light on the role of Nef in the development of HIV-1 disease.

The lack of disease progression in patients whose HIV-1s are *nef*-deleted, defines Nef as a pathogenic factor. Whether Nef acts as a generalized enabler of high levels of replication or is directly pathogenic remains unresolved. In either case it would seem logical to investigate blocking Nef function in order to lessen the severity of HIV-1 disease. Though the idea of Nef as a target for drug intervention in HIV-1 disease has rarely been considered [16,17], Betzi et al. have recently identified the first compounds that target Nef [18]. The major problem is the daunting complexity of Nef's multiple functions. Accordingly, we will discuss four intensely studied Nef activities and assess possible roles for each function in pathogenesis. These are CD4 downregulation, major histocompatibility complex I downregulation, activation of p21-activated protein kinase (Pak2), and enhancement of virion infectivity [19]. Each function is genetically separable from the others and therefore represents a distinct target for inhibiting Nef [20,21]. That each of these four functions is mechanistically distinct implies that an anti-Nef drug will not be able to debilitate Nef in general, but probably block only one or two. This makes it imperative to determine the Nef function most relevant to pathogenesis. In addition, we will discuss the possibility of a radical new approach to viral pathogenesis based on the recent model of simian and human lentivirus pathogenesis being controlled by the downregulation of CD3 by Nef [22]. Finally, we will conclude that an attenuated virus vaccine based solely on a Nef deletion is still remote, and that CD4 downregulation is the most promising target for attacking HIV-1 through Nef.

### Nef and disease progression

Nef was first shown to be a major determinant of primate lentivirus pathogenicity when it was demonstrated that a large deletion in the *nef *gene greatly reduces the severity of simian immunodeficiency virus (SIV) induced disease in rhesus macaques. Furthermore, following intravenous injection of macaques with an SIV encoding a *nef *gene with a premature stop codon, the *nef *open reading frame (ORF) was rapidly restored. This demonstrated that there was significant selective pressure to express the SIV Nef protein [23]. HIV-1 Nef also has a key role in pathogenesis. There are four separate examples of LTNPs infected with *nef*-deleted HIV-1. As indicated above, the best studied is the Sydney Blood Bank Cohort [15]. Infection occurred in the short time frame between the appearance of HIV-1 in Australia and the institution of HIV-1 blood testing. A single donor contributed multiple units of contaminated blood. Red cells or platelets from that blood were given to ten patients with eight of these recipients becoming infected [24]. The high rate of infection is comparable to the rate of transfusion-associated HIV-1 infection in general which is approximately 60% [25]. This is a striking result since the blood contributed by the donor in the SBBC carried low levels of *nef*-defective virus. Clearly, Nef is not required for transmission by blood, but is a crucial factor for disease development. It is important to note the rarity of blood transfusion related infections by *nef*-deleted HIV-1s. Only one other case has been reported- a hemophiliac infected by a Factor VIII preparation contaminated with HIV-1 [12] compared to the 12,000 transmissions through the blood supply in the United States alone [25].

Though Nef is not required for blood to blood transmission it appears to be an important factor for sexual transmission. This is shown by the fact that there are only four reported cases of non-transfusion related infections by Nef-defective virus. These are the donor in the SBBC cohort who was a sexually active homosexual male [11], a homosexual male from Italy [14], and a male who contracted a *nef *defective virus heterosexually in Thailand and then transmitted the virus to his wife [13]. The virus from all SBBC recipients and three of the four just mentioned sexual transmissions exhibit a surprising convergence. They all have two similar defects in the *nef *gene. First, the coding region of Nef from near the initiation codon to near the 5' end of the polypurine tract (ppt) is deleted. Second, there is a large deletion from just downstream of the ppt to the end of Nef but not into the major promoter elements of U3. The simple explanation for these genetic convergences is that the two described regions have no major functions other than to code for Nef, and in the absence of Nef function a slight advantage is accrued to replication by completely deleting them. The one exception was the male who contracted AIDS in Thailand. This subtype E virus exhibited a wide range of Nef sequences from intact to large deletions including the ppt. Blood samples from the time of HIV-1 transmission to his wife are not available [13] to explain how she came to be infected with the double deleted Nef just described.

Death as a result of AIDS has not been observed in any of the people infected by Nef-deleted virus, but in some cases it was apparent that disease was advancing. There are 6 SBBC patients (C49, C64, C135, C54, C98, D36) whose HIV-1 infections have been extensively documented. Three recipients- C49, C64, and C135- lived over 20 years without any sign of disease. Virus was not detectable in blood from these patients, and they exhibited minimal antibody responses [26]. Therefore, these patients are "elite" long term non-progressors. Three additional patients had detectable viral loads and an eventual decline in CD4+ T cells in blood after 17 or more years of being infected. C54 died of non-AIDS causes before the decline in CD4+ T cells necessitated anti-retroviral drugs. C98's CD4+ T cells declined to nearly 200 cells/ml, and received anti-viral therapy for 16 months before dying of non-AIDS causes. The blood donor of the cohort, D36, declined after 18 years to 160 CD4+ T cells/ml and developed HIV-associated dementia [27]. At the point of commencing therapy his plasma HIV-1 RNA was 9900 copies/ml and there were over 750,000 copies/ml in cerebrospinal fluid. One month after receiving therapy plasma viral load was undetectable and CD4+ T cell levels increased [28].

These last three patients are best described as slow progressors (SP). Another SP was the above mentioned hemophiliac infected through a contaminated Factor VIII preparation [12]. This individual was one of 7 LTNPs out of a study group of 128 infected hemophiliacs [29]. PCR screens for full length Nef genes yielded only this patient as having a doubly truncated Nef. For about 10 years post-infection his CD4+ T cell counts were stable, but after another 3 years his CD4+ T cell count fell to 261 and HAART was initiated [30]. An additional case of a LTNP is an Italian homosexual whose CD4+ T cell levels have not altered in 20 years of infection and whose viral loads have been steady at the extremely low value of about 200 copies/ml. As previously mentioned Nef sequences derived from this person's virus contained two deletions in Nef upstream and downstream of the ppt [14]. Nine years later the entire HIV-1 genome from this individual was sequenced. Surprisingly, sequence of the *env *gene, but not *gag*, *pol*, *vif*, *vpr*, *tat *or *rev*, also showed large deletions [14,31]. Large deletions in genes other than *nef *have not been seen in the SBBC [32]. Finally, the husband and wife that are infected with a *nef*-deleted subtype E virus also appear to be LTNPs. They have not shown any signs of disease progression but they may not have been infected longer than 10 years [13].

Summarizing these studies it is evident that *in vivo *Nef is a critical factor in HIV-1 replication, but it is not absolutely necessary. Despite patients infected with *nef*-defective HIV-1 having little or no virus in their blood some did progress towards HIV-1 disease. What percentage of HIV-1 infected individuals have *nef*-deleted virus is difficult to estimate since without disease progression many cases could go undetected. If the percentage were anything other than extremely low, one would certainly expect many more cases to have been uncovered. The same argument applies to the transmission of *nef*-defective HIV-1 sexually. For example, the HIV-1 positive status of the husband and wife pair was revealed as a result of testing during pregnancy [13]. Therefore, it would seem that Nef is not only a pathogenic factor but also a sexual transmission factor.

### Which Nef functions are required for pathogenesis?

Nef is a small protein devoid of enzymatic activity. It is polymorphic in length (200–215 amino acids) with the most common length being 206 [33]. It is myristoylated and mainly localized in the paranuclear region with reduced expression at the plasma membrane. It serves as an adaptor protein to divert host cell proteins to aberrant functions that amplify viral replication [34,35]. Four *in vitro *activities of HIV-1 Nef have been extensively documented. They are: 1) Nef downregulates cell surface levels of CD4 [36-40]; 2) Nef downregulates cell surface levels of major histocompatibility class I (MHCI) molecules [41-45].; 3) Nef mediates cellular signaling and activation [46-49]; and 4) Nef enhances viral particle infectivity by CD4 independent mechanisms [50-55].

Each of these four Nef functions could serve as contributors to Nef's elusive role in replication and pathogenesis. Several reports have suggested the importance of removing CD4 from the surface of infected cells for the production of infectious HIV-1 particles [39,56]. Without this Nef function host cell CD4 can bind to Env during virion budding and interfere with the production of fully infectious particles. Also, Nef's ability to down-modulate MHCI molecules could facilitate HIV-1 immune evasion and thus enhance virus replication [57,58]. A third possible Nef-mediated enhancement of pathogenesis is cellular activation of cell signaling pathways that could enhance replication in partially stimulated T cells. For example, if Nef functions *in vivo *to elevate the activation level of certain partially activated T cell populations then viral production in those cells would be increased [59,60]. Of particular interest in this regard are the memory T cells in the gut that are early targets of HIV-1 and SIV infection, even though they lack expression of classic T cell activation markers [61-63]. Finally, the well documented Nef-dependent enhancement of the infectivity of viral particles would be expected to accelerate the spread of virus *in vivo*. This function of Nef is distinct from the role that CD4 downregulation can play in the production of competent HIV-1 virions. These four Nef functions will now be discussed in greater detail.

#### A. CD4 down-modulation by Nef

The first and most extensively characterized function of Nef is its ability to dramatically reduce the steady state levels of CD4 on the cell surface [38,64])) Human CD4 is downmodulated by Nefs from HIV-1 groups M, N, and O, and simian immunodeficiency virus from chimpanzees (SIV_CPZ_) [65], in multiple mammalian cell types [37,66], and even in Drosophila S2 cells [67]. As mentioned above the major role for this Nef activity may be in overcoming the detrimental effects of high cellular CD4 expression in the producer cell [39,68,69].

**Figure 1:**
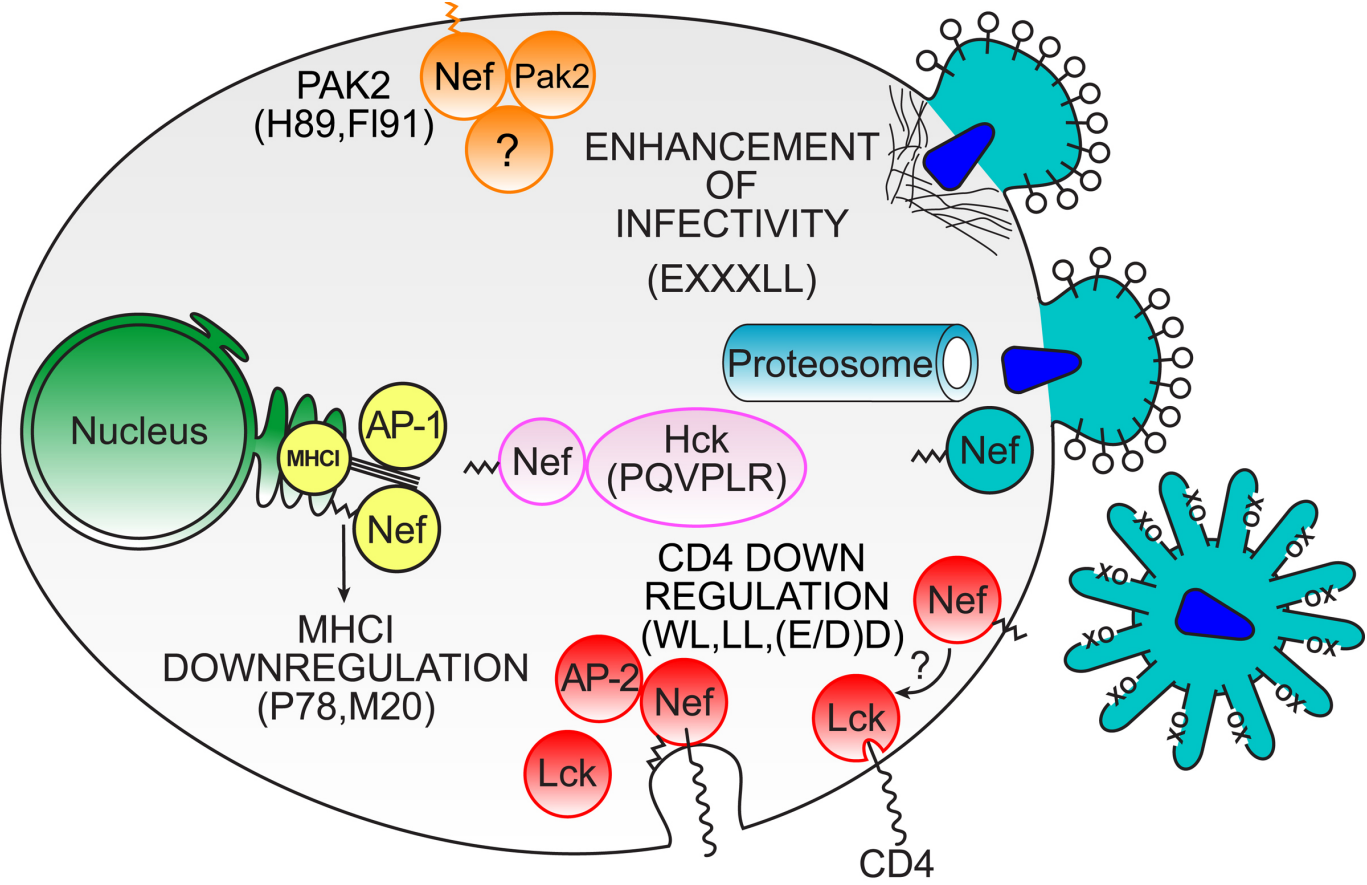
**Diagram illustrating the functions of Nef discussed in the text.***Lower right *(*Red*), Nef removes CD4 from the cell surface. Two processes are shown. To the right Nef is attached to the plasma membrane through its myristoyl group (squiggle) and is detaching Lck from the cytoplasmic tail of CD4. As indicated by "?" both the site and mechanism of this process are unknown and may be indirect. To the left Lck has been disassociated from the cytoplasmic tail of CD4 and Nef is attached to the plasma membrane by its myristoyl group and the cytoplasmic tail of CD4. AP-2 binding facilitates the formation of a clathrin coated pit that leads to the internalization of CD4. *Left *(*Yellow*), Nef downregulates MHCI from the surface of the infected cell. Nef binds to the cytoplasmic tail of MHCI (triple line) and AP-1 in the TGN to divert MHCI from the default pathway to the plasma membrane. *Top *(*Orange*), Nef activates Pak2. The identities of the other protein(s) in the Nef/Pak2 complex are not known as shown by the unidentified protein (?). The cellular site of the activation is also not known though the plasma membrane has been proposed. *Center *(*Pink*), Nef binds to and activates Hck. The central (cytosolic) location of Nef bound to Hck with no attachment of the myristate to a membrane indicates that the activation of Hck is the only Nef function that does not require this post-translational modification. *Upper right *(*Blue*), Nef enhances the intrinsic infectivity of the HIV-1 virion. Three proposed mechanisms that limit HIV-1 infectivity, but are overcome by Nef are presented. The top virion fusing with the cell membrane is attempting to insert the viral core into the target cell but the entry of the core is blocked by cortical actin. The lower virion entering the cell is able to efficiently pass through the cortical actin but is subject to proteosomal degradation upon entry. The extracelluar virion is being prevented from attaching to the target cell by the presence of an unknown protein (X) that prevents Env (O) binding to target cell CD4.

Nef-induced CD4 down-modulation involves the internalization of surface CD4 followed by degradation via the endosomal/lysosomal pathway (Figure 1, *Red*). Consistent with this mechanism Nef localizes to clathrin-coated pits [70] and increases the number of CD4 containing clathrin coated pits [71] Inhibition of lysosomal acidification blocks Nef induced CD4 degradation, without restoring CD4 surface expression [72-74] Moreover, Nef induced CD4 downmodulation is blocked by transdominant-negative dynamin-1 co-expression [75], as well as, pharmacological inhibitors of clathrin coated pit mediated endocytosis [74].

The heterotetrameric clathrin-associated adaptor protein 2 (AP-2) is a key molecular mediator of Nef induced CD4 downmodulation [76], but other aspects of CD4 downregulation remain unclear. Unlike CD4 downmodulation by phorbol esters, Nef-induced downmodulation is independent of the phosphorylation of serine residues in the CD4 cytoplasmic tail [38]. Data suggest that Nef may act as a connector between CD4 and the cell's endocytic machinery [40], by binding the membrane proximal segment of the cytoplasmic domain of CD4 [37,38,77] Furthermore, NMR analysis confirms that the membrane proximal segment of CD4 is necessary for a direct interaction with Nef [78] Nef residues W57 and L58 are predicted by NMR to be critical in this interaction and have also been functionally demonstrated to be important for CD4 downmodulation [79]. The possible significance of this proposed interaction between Nef and the cytoplasmic tail of CD4 is obscured by the fact that it is weak, but the interaction of p56^*lck *^and CD4 is strong and the p56^*lck*^-CD4 complex is not subject to rapid endocytosis [80,81] Further, it is unlikely that Nef binds directly with p56^*lck *^intracellularly [82], even though Nef has been shown to induce endosomal accumulation of Lck [83]. This latter effect of Nef on Lck does not appear to be related to CD4 downregulation since the L164A/L165A mutant of Nef alters the intracellular distribution of Lck but fails to downregulate CD4 [83,84]. An alternate model to the direct binding of Nef to the cytoplasmic tail of CD4 has been proposed by Coleman et al. in which Nef disregulates endosomal trafficking [85].

In contrast to the poorly defined direct interaction of Nef with the cytoplasmic tail of CD4 the direct interaction of Nef with AP-2 has been described in detail [76]. AP-2 binds to the just mentioned dileucine motif in Nef which is found in a structurally flexible loop that extends from amino acids 148 to 180 [86]. The dileucine motif in Nef exhibits a canonical ^160^EXXXLL^165 ^sequence but it is not sufficient to account for the binding of Nef to AP-2. Also required are two acidic residues within the loop, ^174^(E/D)D^175^. Mutation of either the dileucines or the diacidic residues to alanines disables Nef binding to AP-2 in yeast three hybrid assays (Nef/AP-2α/AP-2σ2) and the CD4 downregulation function. In the absence of the diacidic residues there is weak binding by the dileucine motif because of a suboptimal sequence for the XXX residues (i.e. N, T and S). Replacing ^161^NTS^163 ^within the Nef dileucine motif with residues from the AP-2 interacting protein, tyrosinase, gives ^160^ERQPLL^164 ^which even in combination with the ^174^AA^175 ^mutation binds strongly to AP-2. The arrangement of a weak dileucine motif which is apparently stabilized by nearby acidic residues may be peculiar to Nef. This led Lindwasser, et al. to suggest the Nef/AP-2 interaction as a possible target for anti-virals to counter the pathological effects of HIV-1 [76]. The possibility that blocking CD4 downregulation could have a positive impact on HIV-1 pathogenesis is supported by the example of an LTNP infected by a HIV-1 with a uniquely defective Nef. Carl et al. reported a non-progressor (12 years without a decline in CD4+ T cells, but relatively high viral loads of 15,000 to 55,000 copies/ml) with a small deletion in Nef and a compensating duplication [87]. The virus in this patient had a deletion of 36 base pairs (amino acids 26–37) and a 33 base pair duplication (amino acids 43–53). In vitro studies demonstrated that the deletion by itself inactivates CD4 downregulation, enhancement of infectivity, MHCI downregulation, and partially destabilizes the protein. Incorporating the duplication into the deletion bearing Nef gave a partially functional protein that had restored enhancement of infectivity, MHCI downregulation, and protein expression but remained defective for CD4 downregulation. The suggestion from this one patient is that an HIV-1 lacking a Nef functional for CD4 downregulation is greatly reduced in its pathogenic potential. Therefore, Nef-mediated CD4 downregulation appears to be a potential target for anti-viral intervention, except that the flexible structure of the loop containing ^160^EXXXLL^165 ^and ^174^(E/D)D^175 ^may not allow for the modeling of small molecules with high affinity and specificity [18]. Therefore, the potentially unique Nef-binding surface of AP-2 may be a better target.

#### B. MHC class I down-modulation by Nef

Another well conserved property of Nef is its ability to downmodulate MHC class I molecules [44]. As Nef is expressed early after infection, Nef induced downmodulation of MHC class I molecules could enable the infected cell to evade destruction by the immune system during active viral replication. In support, it has been demonstrated that Nef expression reduces the susceptibility of HIV infected cells to cytotoxic T lymphocyte (CTL) mediated lysis *in vitro *[57,58]. Therefore, determining the mechanism by which Nef downregulates MHCI has received a high priority. Early aspects of this field have been reviewed [88].

A long standing model proposed by Thomas and co-workers [89,90] has been recently revised [41]. In this model Nef initiates MHCI downregulation by interacting with PACS-2. The Nef/PACS-2 complex localizes to the *trans*-Golgi network (TGN) where PACS-2 is displaced and Nef binds to a *src *family kinase (SFK). The SFK would then bind and phosphorylate ZAP70/Syk on tyrosine enabling ZAP70/Syk to bind the SH2 domain of phosphatidylinositide 3-kinase (PI3K). The resulting activation of PI3K would lead to elevated PIP_3_, stimulation of the guanine nucleotide exchange factor ARNO, and GTP loading of ARF6. At this point the rate of MHCI endocytosis is accelerated. For the increased rate of MHCI internalization to be effective in reducing MHCI cell surface levels Nef must also block the recycling of MHCI back to the plasma membrane. The revised model does not define an interaction between MHCI and Nef, but the suggestion was made that Nef induces PACS-1 to interact with the cytoplasmic tail of MHCI [41]. However, a ternary complex between Nef, the cytoplasmic tail of MHCI and AP1 has been recently demonstrated separately by the Collins and the Guatelli laboratories [43,45]. This complex appears to activate a cryptic tyrosine sorting signal in the cytoplasmic tail of MHCI and diverts newly synthesized MHCI molecules from their transit to the plasma membrane to an internal compartment in the paranuclear region [43,45,91]. Nef appears to be acting as a facilitator since the cytoplasmic tail of MHCI does not bind to AP-1 [43,45]. How the model of Thomas and co-workers can be adapted to include this complex is as yet unresolved (Figure 1, *Yellow*). It is interesting to note that this ternary complex engages Nef in a novel interaction with MHCI cytoplasmic tail and AP-1 which makes it potentially appealing for targeting by an anti-viral.

However, it should be noted that Nef does not render infected cells completely protected from immune surveillance as there is a strong CTL response to HIV antigens [92]. Therefore, it appears that the downregulation of MHCI by Nef fails to block the cytotoxic T cell response to the virus, but the immune response is either misdirected or HIV-1 is able to escape by mutation or both [93]. At this point the concept of viral evolution and its relationship to viral pathogenesis should be considered. That there are constraints placed on the virus by the cytotoxic T cell response is clear if the virus mutates to avoid the response [94,95]. This does not necessarily imply that the targeted HIV-1 or the HIV-1 bearing escape mutation(s) are different in pathogenic potential. In fact, Brumme et al. [94] found an inverse relationship between the number of apparent escape mutations in Nef and the level of CD4^+ ^T cells in the blood. In other words, virus with a Nef containing 11 or more escape mutations was more pathogenic than virus with a Nef containing 0–2 mutations. This distressing finding likely reflects Nef having successfully evolved to readily side-step the vast majority of CTL responses. Nef has 63 very highly conserved residues out of 206 (99% identity), but they are scattered throughout the protein so that no more than five are in a row [33]. As a result, susceptible epitopes in Nef mutate at variable residues to effectively escape CTLs, but Nef function is not affected.

A relatively small number of HLA epitopes in HIV-1 genes other than Nef have been reported that do involve escape mutations that reduce virulence. These epitopes have been suggested as the basis for a therapeutic vaccine [95]. Although Nef amino acid sequences are doubtful contributors to the proposed vaccine an inhibitor of Nef's ability to downregulate MHCI could enhance the effectiveness of such a vaccine. In this regard the Italian male infected with a virus lacking *nef *subsequently evolved a virus with both a deleted *nef *and *env*. Calugi et al [31] interpreted this finding as an inability of the Nef deleted virus to protect itself from CTL attack. Unfortunately, this patient appears to be unique as the SBBC researchers did not find evidence of the development of deletions in other genes [32].

#### C. Cellular activation and signaling by Nef

Disease progression may be directly associated with T cell activation [96,97]. It is also possible that Nef may regulate cellular activation through several kinases including Pak2 [48,49]. and Hck [82,98]. Pak2 is the best characterized Nef-activated kinase. It has been demonstrated that Nef activation of Pak2 leads to merlin phosphorylation at serine 518 though it has yet to be demonstrated that HIV-1 infection is in anyway dependent on merlin phosphorylation [46]. The obvious suggestion of this result that Nef regulates the actin cyotskeleton function is appealing, but the mechanism is controversial [99-102].

Substantial agreement exists that Nef forms a complex with Pak2 (Figure 1, *Orange*) [65,100,103,104]. Nef not only complexes with Pak2 but also induces Pak2 activation [49,105] The ability of Nef to activate Pak2 in multiple HIV-1 subtypes suggests a key role for this Nef function [33,106]. An interaction domain that is responsible for Pak2 activation has been observed to include residues 89 and 191 [33]. Lesser contributions are made by residues 85 and 188 [103,106,107]. Remarkably, H89 and F191 are highly conserved in subtype B Nefs, but in subtype E Nefs F89 and R191 are highly conserved instead [33]. Since subtype E Nefs are active for Pak2 activation it appears that at least two different interaction domains are functional in HIV-1 Nefs. By substituting all four just mentioned residues in a subtype B Nef with residues that predominant in subtype E Nefs (L85F, H89F, R188A, and F191R) the subtype E Pak2 interaction surface can be created in a subtype B background. The quadruple mutant is fully functional though intermediate forms are generally defective [33]. The significance of these alternate Pak2 activation domains remains to be determined. However, the presence of different structures to achieve the same function strongly suggests that maintaining the ability to activate Pak2 enhances viral fitness. The importance of Pak2 activation has been questioned on the basis of *ex vivo *experiments [108]. One possibility is that Pak2 activation may be important for transmission or early in infection. However, only limited evidence currently exists to support this hypothesis [21]. Regardless, the structural fluidity of Nef's Pak2 interaction surface could make this Nef interaction difficult to target with anti-virals. Investigations to uncover the common features of the alternative activation domains may clarify these issues.

Nef also activates the myeloid lineage specific tyrosine kinase, Hck (Figure 1, *Pink*). Co-expression of Nef and Hck in Rat-2 fibroblasts leads to cellular transformation [109]. Moreover, Nef tightly binds to the Hck SH3 domain *in vitro *and activates its kinase activity [110]. In Rat-2 cells enforced dimerization of Nef enhances Hck activation [98]. Nef has also been shown to modestly activate endogenous Hck and, in turn, the Stat3 transcription factor in myeloid cells [111]. Interestingly, Hck is the only cellular activity of Nef known to not require Nef myristoylation [111]. The Hck/Nef interaction is mediated by an SH3 binding domain in Nef ^72^PQVPLR^77 ^which may be too similar to cellular SH3 interactions to be readily targeted by an anti-viral. On the other hand, one distinctly virus-specific interaction would be Nef dimerization which may be required for Hck activation intracellularly. In fact, Nef spontaneously forms dimers and trimers [112]. This may occur when the myristate moiety is buried in a cellular membrane. Disengagement from membrane appears to result in the myristoylated N-terminus of Nef self-associating with a hydrophobic patch on the surface of the structured core of Nef. In this latter conformation Nef is a monomer. Therefore, Nef lacking its N-terminal myristate may dimerize and activate Hck. The relevance of Hck activation for pathogenesis is unknown, but if Nef dimerization and trimerization are of pathological significance it could be a novel target for anti-virals.

#### D. Enhancement of HIV-1 infectivity

Early work on this topic has been previously reviewed [88]. Currently, there are three models of how Nef enhances the infectivity of HIV-1 as measured in single infection assays [50,54,113,114]. In these assays virus is produced in cells that do not express CD4 and the normalized infectivity is determined on indicator cell lines. Therefore, this effect represents increased infection efficiency for HIV-1 virions, and is distinct from Nef-dependent enhanced particle egress from infected macrophages [115]. Campbell et al. noted that the disruption of the actin cytoskeleton in the target cell complemented the defect in infectivity in Nef minus virions (Figure 1, *Blue*). These authors concluded that cortical actin represents a barrier to infection and that the expression of Nef in the producer cell is able to overcome this barrier [54]. In an alternate model Pizzato et al. suggest that there is an unknown cellular protein other than CD4 that blocks the function of Env in the virion. Nef enhances infectivity by downregulating this unknown protein in the producer cell and blocking its incorporation into the virion [50]. It is interesting to note that the Nef dileucine motif (^164^LL^165^) that is required for CD4 downregulation is also required for enhancement of infectivity [116]. However, the diacidic motif (^174^DD^175^) that is necessary for Nef to bind AP-2 is not. This genetically distinguishes enhancement of infectivity from CD4 downregulation. In this case it appears that Nef's canonical dileucine binding motif involving E160 (^160^EXXXLL^165^) associates with AP-1 and AP-3 [85,116,117]. It is not yet known if these results are related to the above mentioned results of Pizzato [50]. Finally, Nef may protect the viral core from post-fusion degradation to allow reverse transcription to proceed [113,114]. This mechanism is consistent with the active degradation of HIV-1 virions that occurs upon entry [118]. Despite the attractiveness of a drug that reduces the inherent infectivity of HIV-1 virions the prospects for inhibiting Nef-mediated enhancement of infectivity are presently remote.

#### E. Downregulation of CD3 as a mechanism of attenuating viral pathogenesis

Schindler et al. have proposed a surprising explanation for the lack of pathologic effects of most primate lentiviruses in their hosts in contrast to the virulence of HIV-1. Specifically, it was proposed that most simian viruses self-limit their inherent pathogenicity [22]. For example, sooty mangabeys do not develop AIDS from their own SIV [119,120]. Schindler et al suggest this is the result of the SIV from sooty mangabey (SIV_SM_) having a Nef that downregulates CD3 which prevents activation of the infected T cell and subsequent activation induced cell death. The authors further propose that the downregulation of CD3 evolved as a mechanism to maintain virus persistence in the presence of an intact host immune system. The CD3 downregulation function was lost in chimpanzee immunodeficiency virus (SIV_CPZ_) prior to the infection of humans. Since HIV-1 Nef does not downregulate CD3 humans progress to AIDS as hyperactivation slowly destroys the immune system. The therapeutic implications of this concept are staggering since one must assume that during the course of natural SIV_SM _infection there are mutations in *nef *that abrogate CD3 downregulation. The unanswered question is how these potentially pathogenic SIV_SM _mutants are suppressed by the non-pathologic virus? If such a mechanism were to be demonstrated it may be possible to produce an HIV-1 with a Nef that downregulates CD3 and therefore a dominant, non-pathogenic virus.

A distinctly different explanation of non-pathogenicity is that the sooty mangabey itself has a special mechanism for suppressing the progression to AIDS not the virus [121]. This would explain the fact that rhesus macaques develop AIDS when directly infected with blood from an infected sooty mangabey, but infection of virus-free sooty mangabeys does not [119]. In other words, rhesus macaques die even though the infecting SIV_SM_'s Nef is downregulating CD3. An additional example of SIV_SM _being pathogenic when a species barrier is crossed is SIV_SM _causing AIDS in a black mangabey [122]. Two additional lineages of SIV including African green monkey (SIV_AGM_) and sun-tailed monkey (SIV_SUN_) cause AIDS in pig-tailed macaques [123,124], but not that from Sykes' monkey (SIV_SYK_) [125]. Like Nef from SIV_SM _the Nefs from SIV_AGM_, SIV_SUN_, and SIV_SYK _all downregulate CD3 [22,126]. It should be further noted that the non-pathogenicity of SIV_SM _is not absolute in sooty mangabeys. Ling et al. have reported a 21 year old sooty mangabey which developed AIDS [121]. These investigators suggest that the evolutionary adaptation to SIV_SM _is one of delayed progression beyond the usual lifespan of the animal which for sooty mangabeys is under 20 years. In the future it will be important to demonstrate SIV_SM _and/or SIV_AGM _specifically defective in CD3 downregulation are significantly pathogenic in their natural hosts.

Schindler et al. have generalized the hypothesis that CD3 downregulation prevents lentivirus pathogenesis by investigating human immunodeficiency virus 2 (HIV-2) [22]. HIV-2 is a zoonotic virus derived from sooty mangabeys [127]. It has reduced pathogenicity relative to HIV-1 overall, but progression to AIDS can occur [128]. Consistent with reduced pathogenicity HIV-2 Nefs do downregulate CD3 [129], but in the cases in which HIV-2 infection has progressed to AIDS one would expect that Nefs derived from these patients would not be functional for CD3 downregulation. This is not the case for Nefs from HIV-2_ROD_, HIV-2_BEN_, and HIV-2_CBL23 _which all came from symptomatic patients and all downregulated CD3 [129]. The pathogenic phenotype of HIV-2 can also be observed in rhesus macaques [130,131]. To demonstrate that CD3 downregulation can block disease progression in humans it will be important to thoroughly investigate the relationship between HIV-2 AIDS and CD3 downregulation.

That a given species may be resistant to its own lentivirus without involvement of CD3 downregulation is clearly demonstrated by the non-pathogenic nature of SIV_CPZ_. In the case of chimpanzees the downregulation of CD3 cannot serve as a mechanism for non-pathogenicity since SIV_CPZ _Nef does not downregulate CD3 [22]. Humans lack the ability that chimps have to resist the chimpanzee virus and develop AIDS. Finally, there is evidence that virus with the capacity to downregulate CD3 can not delay HIV-1 pathogenesis. This is suggested by the cases of dual HIV-1/HIV-2 infection. Despite the ability of HIV-2 Nef to downregulate CD3 these patients suffer the greater virulence of HIV-1 [132]. Therefore, it would appear that the non-pathogenic phenotype attributed to Nefs that downregulate CD3 is not dominant over the pathogenic phenotype in humans.

Overall, we conclude that at present there are minimal prospects for therapeutic insights resulting from the attribution of HIV-1 pathogenicity to the inability of HIV-1 Nef to downregulate CD3. If this proposal is to be developed further, it will be necessary to molecularly define the structural correlates of CD3 downregulation. Then the determination could be made if HIV-1 with a Nef modified to downregulate CD3 has reduced pathogenesis in the humanized mouse model [133]. Alternatively, the pathogenesis of HIV-2s with and without the ability to downregulate CD3 could be evaluated for virulence in humanized mice.

### Attenuated vaccines

The mechanism of adaptation to SIV_SM _by sooty mangabeys suggested by Ling et al. [121] is analogous to the approach taken for highly active antiretroviral therapy. Ideally, SIV_SM _and HIV-1 infections are restrained to be sufficiently chronic and long-lasting that progression to AIDS fails to occur in the life time of the host. This approach is decidedly less desirable than an effective vaccine. Unfortunately, standard vaccine approaches are proven failures. The viral Env has evolved to a highly complex structure that in its native form resists antibody recognition [134]. Broadly neutralizing antibodies (BNAB) do develop during HIV-1 disease that recognize highly conserved epitopes in Env [10], but Env escape variants develop [135,136]. The best hope for preventing HIV-1 infection would be if an attenuated vaccine were to yield BNAB prior to infection. This would allow the immune system to attack the virus early in infection when it is most vulnerable. One positive aspect of the study of the SBBC is that the slow progressors D36, C54, and C98 were able to produce BNAB [15,26]. Unfortunately, these are the three patients that demonstrated disease progression. The LTNPs C49, C64, and C135 had little or no BNAB activity in their blood. So we don't know if C49, C64, or C135 were "immunized" against HIV-1. Therefore, despite massive efforts to understand the "natural immunization" of SBBC patients with *nef*-deleted virus there is little evidence that this type of attenuated virus can be effectively or even safely employed.

The progression of D36, C54, and C98, and the failure of C49, C64, and C135 to maintain BNABs have been rationalized by the threshold hypothesis [132]. The divergent patient responses to being exposed to an attenuated virus result from multi-factorial host-virus dynamics. A seroconversion threshold may be reached by a certain level of viral replication, but vaccine protection fails to develop. Conversely, the vaccine threshold may be surpassed resulting in disease development if viral replication is too active. Thus, development of an attenuated virus that can with virtual certainty yield the correct level of replication for non-pathogenicity but still induce a significant and long-lived immune response in the majority of recipients may not be possible. In 1994 the World Health Organization emphasized the importance of determining the optimal combination of genes that could be deleted to ensure safety of an infectious attenuated HIV-1 vaccine [137]. The complexity of defining such a combination of genes is indicated by the report of Churchill et al. [138] that further characterized the two SBBC cohort members that progressed to the point of HAART treatment. The virus from slow progressor, D36, was partially defective in *rev*, but slow progressor C98 had a fully functional *rev*. Therefore, the a second defective gene in addition to *nef *may not reliably further attenuate the virus. As knowledge of the functions of Vif and other HIV-1 accessory proteins grows superior schemes involving combinations of inactivated genes may become apparent for attenuating HIV-1.

## Conclusion

Anti-HIV-1 drugs rapidly become ineffective unless administered in multi-drug combinations. More drugs attacking multiple aspects of the viral replication cycle and especially transmission are needed to treat and prevent HIV-1 infection. The enzymatic activities reverse transcriptase and protease have been attacked by anti-virals, but developing drugs that target novel interactions between viral and host cell proteins will be more difficult. Nonetheless, given the relentless nature of HIV-1 all reasonable possibilities should be considered. For Nef the downregulation of CD4 appears to be the most promising function to disrupt by an anti-viral. Evidence in hand indicates this approach has the distinct potential to blunt HIV-1 pathogenesis. MHCI downregulation is more problematic despite a novel Nef/host cell protein-target interaction because blocking this function may not significantly impact pathogenesis. However, an anti-Nef drug targeting MHCI downregulation may enhance the ability of a CTL-directed therapeutic vaccine. Pak2 activation may be particularly difficult to target and enhancement of virion infectivity is insufficiently understood to even know what to target.

In a recent development Betzi et al. have identified drug-like compounds (D1 and DCL27) that interact with the SH3 binding domain of Nef [18]. D1 has been observed to interfere with the binding of Hck to Nef, weakly inhibit MHCI downregulation, but have no effect on CD4 downregulation. The effect of D1 on MHCI downregulation may be the result of the proximity of the SH3 binding domain ^72^PQVPLR^77 ^to P78 which is crucial for formation of the ternary complex formed between Nef, the cytoplasmic tail of MHCI, and AP-1 [43,45]. Whether D1 binds to cellular SH3 binding domains remains to be determined. Like progress against HIV-1/AIDS, progress in understanding Nef, has been tedious and difficult, but there is no option to continuing to acquire more knowledge about the virus and its proteins.

## Competing interests

The authors declare that they have no competing interests.

## Authors' contributions

Both authors contributed to the writing and editing of the manuscript.
